# Comparative Prefrontal Multimodal Physiological Signatures Under Active- and Passive-Fatigue-Inducing Simulated Driving Paradigms

**DOI:** 10.3390/brainsci16050508

**Published:** 2026-05-08

**Authors:** Feiyang Zhang, Dequan Fang, Shiji Yuan, Huaizhi Tang, Xiao Liang, Shuai Wang, Kang Ma, Dezhi Zheng, Shangchun Fan

**Affiliations:** 1 School of Instrumentation and Optoelectronic Engineering, Beihang University, Beijing 100191, China; 1417zfy@buaa.edu.cn (F.Z.);; 2 School of Interdisciplinary Sciences, Beijing Institute of Technology, Beijing 100081, China; 3 School of Computer Science and Engineering, Beihang University, Beijing 100191, China; 4 School of Computer Science and Engineering, Beijing Institute of Technology, Beijing 100081, China; kangx.ma@gmail.com; 5 MIIT Key Laboratory of Complex-Field Intelligent Sensing, Beijing Institute of Technology, Beijing 100081, China; 6 State Key Laboratory of Environment Characteristics and Effects for Near-Space, Beijing Institute of Technology, Beijing 100081, China

**Keywords:** mental fatigue, prefrontal physiological signatures, electroencephalography, functional near-infrared spectroscopy, pulse rate variability, simulated driving

## Abstract

**Highlights:**

**What are the main findings?**

- Two simulated driving paradigms designed to induce active and passive fatigue showed distinct stage-dependent prefrontal multimodal physiological signatures.
- Active fatigue showed more continuous EEG changes and clearer averaged hemodynamic modulation, whereas passive fatigue showed weaker averaged hemodynamic effects and clearer pulse-variability accumulation.

**What are the implications of the main findings?**

- A three-level fatigue framework helps distinguish early, late, and cumulative physiological changes that are not captured by binary fatigue classification.
- Multimodal prefrontal monitoring combining EEG, fNIRS, and pulse-related measures may support graded fatigue assessment and more refined fatigue monitoring in driving.

**Abstract:**

**Background/Objectives**: Mental fatigue during driving can arise under different task conditions and typically progresses from mild to severe states. Active fatigue is usually linked to cognitively demanding driving, whereas passive fatigue is associated with prolonged monotonous driving. However, studies on multilevel mental fatigue remain scarce, and direct comparisons of prefrontal multimodal physiological responses to active and passive fatigue are still limited. The objective of this study is to characterize and compare the prefrontal multimodal physiological signatures across three fatigue levels under two simulated driving paradigms designed to induce active and passive fatigue. **Methods**: Eleven healthy participants completed two simulated driving tasks designed to induce active and passive fatigue. Physiological data were recorded using a self-developed prefrontal EEG-fNIRS system, and pulse-related signals were derived from the hemodynamic measurements. Based on subjective and objective indicators, fatigue was classified into non-fatigue (NonF), moderate fatigue (ModF), and severe fatigue (SevF). **Results**: In the active-fatigue-inducing paradigm, significant changes in prefrontal EEG and hemodynamic already emerged from NonF to ModF; for example, the EEG β/(θ + α) power ratio increased from 0.973 to 1.157 (*p* < 0.001) and the normalized mean deoxyhemoglobin feature increased from −0.06 to 0.09 (*p* < 0.001). In the passive-fatigue-inducing paradigm, EEG changes became prominent mainly from ModF to SevF, with β/(θ + α) power ratio decreasing from 0.806 to 0.761 (*p* < 0.05). Pulse rate variability showed increasing trends in both paradigms. **Conclusions**: These findings suggest that the two simulated driving paradigms were associated with distinct prefrontal electrophysiological, hemodynamic, and autonomic evolution patterns across three fatigue levels, supporting graded fatigue assessment and multimodal fatigue monitoring in driving.

## 1. Introduction

Mental fatigue is a psychophysiological state that emerges during prolonged task performance and is typically accompanied by reduced alertness, impaired cognitive efficiency, and deteriorated behavioral performance [1,2]. In driving, mental fatigue is a major safety concern because safe vehicle operation depends on sustained vigilance, rapid decision making, and continuous sensorimotor coordination [3,4,5,6]. As fatigue develops, drivers may exhibit slower reactions, reduced attentional control, impaired judgment, and diminished responsiveness to unexpected events, thereby increasing the risk of unsafe driving and traffic accidents [3,4,5,6]. Accordingly, identifying reliable physiological markers of driving-related mental fatigue has become an important topic in neuroscience, neuroergonomics, human factors, and intelligent transportation research [2,7,8,9,10,11].

Driving-related mental fatigue can arise under different task conditions and should not be treated as a unitary phenomenon [3,12,13,14,15]. In general, active fatigue is usually linked to cognitively demanding driving conditions that require continuous effort, frequent responses, and sustained executive control. In contrast, passive fatigue is more likely to develop during prolonged monotonous driving with low stimulation and reduced task engagement [3,12,13,14,15]. This distinction is theoretically important because the two fatigue types are associated with different workload characteristics and may therefore present different physiological manifestations and intervention needs [12,13,14,15]. It is also practically relevant because real driving includes both demanding and monotonous conditions, and increasing vehicle automation may further increase drivers’ exposure to passive-fatigue-inducing situations [13,14]. In addition, mental fatigue usually develops progressively rather than appearing abruptly, making it necessary to investigate fatigue not only as a binary state but also as a graded process from lower to higher levels [1,2,16].

A growing body of work has investigated physiological markers of mental fatigue using electroencephalography (EEG), heart-related measures, and functional near-infrared spectroscopy (fNIRS) [2,7,8,9,10,11,17,18]. EEG is widely regarded as one of the most informative modalities because it directly reflects cortical activity and is sensitive to changes in vigilance, attention, and cognitive control [8,17,19,20,21,22]. Typical fatigue-related EEG findings include alterations in alpha, theta, beta, and delta activity, although the direction and timing of these changes are not always consistent across studies [8,17,19,20,21,22,23]. fNIRS provides complementary information by monitoring cortical hemodynamic responses and has become increasingly valuable for fatigue research in ecological or wearable settings [9,10,11,18,24,25,26,27]. This is particularly relevant for the prefrontal cortex, which plays a central role in executive regulation, effort allocation, and attentional control during driving [4,10,24,25]. Heart- or pulse-related measures provide an additional autonomic perspective and have also been incorporated into fatigue assessment studies [2,22,28]. These modalities index electrophysiological, neurovascular, and autonomic processes from different perspectives. Therefore, multimodal approaches may provide a more complete characterization of mental fatigue than single-modality studies [10,18,24,25,26,27,28,29,30].

Despite this progress, several limitations remain. First, many previous studies have focused on binary comparisons such as fatigue versus non-fatigue, whereas multilevel or graded fatigue assessment remains relatively scarce [1,2,16]. However, mental fatigue is inherently progressive, and a multilevel framework is more suitable for capturing its evolution and for supporting practical monitoring systems [1,2,16]. Second, many driving studies have investigated either monotonous driving or demanding driving alone, while direct comparisons between active and passive fatigue remain limited [3,12,13,14,15]. Recent work has explicitly noted that many existing biomarkers have primarily been validated under monotonous conditions. This raises questions about their applicability to cognitively demanding driving scenarios [3]. Third, although multimodal physiological sensing is increasingly advocated, comparative studies specifically addressing prefrontal multimodal physiological responses to active and passive fatigue are still insufficient, particularly when EEG, fNIRS, and pulse-related measures are integrated within a unified framework [3,9,10,11,18,24,25,26,27,28,29,30]. Consequently, it remains unclear whether active and passive fatigue should be understood simply as different intensities of the same physiological process or as distinct fatigue processes with different multimodal evolution patterns.

To address these gaps, the present study characterizes prefrontal multimodal physiological signatures across three fatigue levels. These signatures were compared under two simulated driving paradigms designed to induce active and passive fatigue. The main contributions of this work are as follows:

- A three-level mental fatigue grading strategy was proposed by combining coarse classification based on subjective self-ratings with refined subdivision based on facial performance, allowing for mental fatigue to be categorized into non-fatigue, moderate fatigue, and severe fatigue;
- Fatigue-related changes in prefrontal multimodal physiological features, including EEG, fNIRS, and pulse rate variability (PRV), were statistically characterized across different fatigue levels, and features showing significant differences with fatigue progression were identified;
- Differences in prefrontal multimodal physiological patterns under active- and passive-fatigue-inducing driving paradigms were compared, revealing distinct electrophysiological, hemodynamic, and autonomic signatures under different fatigue-induction conditions.

## 2. Materials and Methods

### 2.1. Participants

Eleven healthy male volunteers participated in this study. All participants held valid driving licenses. Their ages ranged from 22 to 31 years, with a mean age of 24.7 ± 4.7 years. All participants had normal or corrected-to-normal vision and reported no history of neurological, psychiatric, cardiovascular, or sleep-related disorders. Only male participants were included to reduce inter-subject variability and improve sample homogeneity in this preliminary comparison of active and passive fatigue. All participants provided written informed consent prior to participation. Before participation, all participants were asked to report medication use, and none reported the use of medications that might affect central nervous system or autonomic function during the experiment. Participants were also instructed to avoid alcohol, caffeine, nicotine, and other stimulants before the experimental sessions.

### 2.2. Experimental Design and Protocol

**Figure 1:**
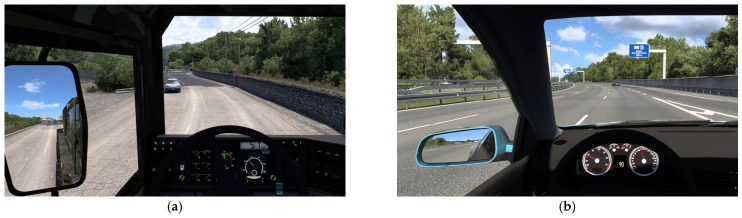
Representative driving scenes and corresponding routes of the two simulated driving paradigms. (**a**) Active-fatigue driving scenario, in which participants drove a truck tractor without a trailer on a circular route around Corsica, under cognitively demanding road conditions characterized by narrow pavement and frequent curves. (**b**) Passive-fatigue driving scenario, in which participants drove a passenger car on the Munich–Warsaw–Leipzig route under prolonged monotonous highway conditions characterized by wide pavement and few curves. The active-fatigue paradigm was designed to impose a relatively high cognitive workload, whereas the passive-fatigue paradigm was designed to impose a relatively low cognitive workload.

A simulated driving environment was constructed using a driving simulator (51 Sim, Shanghai, China) and the commercial driving software Euro Truck Simulator 2. Two driving paradigms with different cognitive workload levels were designed to induce active fatigue and passive fatigue, respectively. Representative experimental scenes of the two paradigms are shown in Figure 1, and their detailed configurations are summarized in Table 1. In the active paradigm, participants drove under relatively complex road conditions that required sustained attention, frequent decision making, and continuous steering and pedal control, thereby imposing a high cognitive workload. In the passive paradigm, participants drove under prolonged monotonous highway conditions and were instructed to use cruise control whenever possible, such that most of the time was spent monitoring the driving environment with only occasional responses, thereby imposing a relatively low cognitive workload.

**Figure 2:**
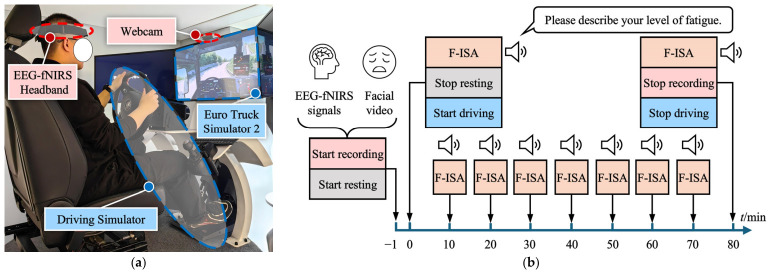
Experimental setup and procedure of the simulated driving fatigue paradigm. (**a**) Experimental setup of the simulated driving task, including the driving simulator hardware, the Euro Truck Simulator 2 interface, the self-developed prefrontal EEG-fNIRS headband, and the webcam used for facial video recording. (**b**) Schematic illustration of the experimental procedure. The task included a resting period followed by continuous driving, during which fatigue instantaneous self-assessment (F-ISA) scores were verbally reported every 10 min. Prefrontal EEG-fNIRS signals and facial videos were synchronously recorded throughout the experiment.

Before the formal experiment, each participant completed a practice session using the same simulator interface. Familiarization was confirmed by the experimenter when the participant could smoothly operate the steering wheel, pedals, and driving interface, maintain lane position, and complete a short practice drive without obvious operational errors or difficulty. Each participant completed one active-fatigue task and one passive-fatigue task, with an interval of at least 24 h between the two sessions to minimize carryover effects. Before each session, participants were asked to confirm that they had sufficient sleep the night before and were in a normal physical and mental state. To reduce potential circadian influences, all experiments were initiated at approximately 3:00 p.m. The procedural structure of the two tasks was identical, as illustrated in Figure 2. Each task lasted 81 min in total, including a 1 min resting period followed by 80 min of continuous driving. During the driving period, participants were asked to verbally report the fatigue instantaneous self-assessment (F-ISA) score every 10 min. Throughout the experiment, prefrontal EEG and fNIRS signals were synchronously recorded using the self-developed EEG-fNIRS headband, and facial videos were simultaneously collected for subsequent fatigue grading.

### 2.3. Mental Fatigue Assessment and Three-Level Labeling Strategy

Mental fatigue was treated as a graded and progressively evolving process rather than a binary state. To characterize its temporal development during simulated driving, a three-level labeling strategy was adopted, in which mental fatigue was categorized into non-fatigue (NonF), moderate fatigue (ModF), and severe fatigue (SevF).

#### 2.3.1. Subjective Fatigue Assessment

During the driving period, participants were asked to verbally report the F-ISA score every 10 min. The F-ISA scale consisted of five fatigue levels: 1 (very low), 2 (low), 3 (medium), 4 (high), and 5 (very high). These scores were used to provide a coarse description of the temporal progression of mental fatigue in both the active- and passive-fatigue tasks.

At each assessment time point, the fatigue level was preliminarily determined according to the F-ISA score. Specifically, an F-ISA score of 1 or 2 was regarded as NonF, a score of 3 was regarded as ModF, and a score of 4 or 5 was regarded as SevF. For the time interval between two adjacent F-ISA reports, if the fatigue levels at the beginning and end of the interval were identical, the entire interval was assigned to the same fatigue level. If the two levels differed, the interval was regarded as a transition segment, indicating that a fatigue-level change occurred within that period.

#### 2.3.2. Facial-Video-Based Behavioral Measures

To refine fatigue transitions within transition segments, fatigue-related facial behaviors were quantified from the recorded facial videos. Two indicators were extracted: the percentage of eyelid closure (PERCLOS) and yawning duration. PERCLOS was defined as the ratio of eye-closure time to the total observation time within a given period. These indicators were calculated based on the open-source Driver-State-Detection project (https://github.com/e-candeloro/Driver-State-Detection) (accessed on 17 June 2024).

PERCLOS was computed once every 8 s, whereas yawning behavior was evaluated every 0.1 s. To reduce false detections, a yawn was considered valid only when its duration exceeded 1.5 s. These facial measures were used to identify abrupt behavioral changes associated with fatigue progression and thereby refine the timing of fatigue-level transitions.

#### 2.3.3. Three-Level Fatigue Label Refinement

Within each transition segment, the fatigue level was assumed to change when a distinct shift in facial behavior occurred. The transition time points were determined according to the following rules.

If the fatigue level increased by *N*_up_ levels between the beginning and end of a transition segment, the corresponding transition points were determined from the earliest fatigue-related facial changes. Specifically, the time points corresponding to the *N*_up_ largest first-order derivatives of the PERCLOS time series and the *N*_up_ earliest valid yawning events were first identified and then manually verified against the facial videos. Among these candidate time points, the earliest *N*_up_ time points were selected as the final transition points.

If the fatigue level decreased by *N*_down_ levels within a transition segment, candidate transition points were first identified from the *N*_down_ smallest first-order derivatives of the PERCLOS time series and then manually verified against the facial videos. Yawning events were not used for downward transitions because yawning is more closely associated with fatigue accumulation than with fatigue recovery.

By combining the coarse temporal segmentation provided by F-ISA with the refined transition timing identified from facial behavior, the entire experimental duration was finally partitioned into NonF, ModF, and SevF periods. These labeled periods were subsequently used as the basis for feature window segmentation and physiological analysis.

### 2.4. Neurophysiological Data Collection and Processing

#### 2.4.1. Prefrontal EEG-fNIRS System and Signal Acquisition

**Figure 3:**
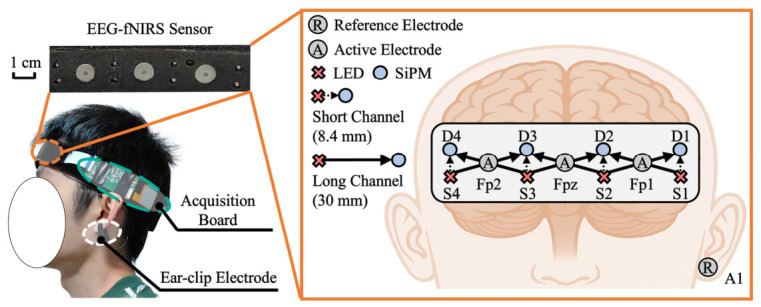
Self-developed prefrontal EEG-fNIRS headband and signal acquisition system.

Physiological signals were acquired using a self-developed prefrontal EEG-fNIRS headband, which consisted of a prefrontal EEG-fNIRS sensor unit, an acquisition board, and a battery, and communicated with a custom upper computer interface via Wi-Fi, as shown in Figure 3. The system was developed based on our previous work on a flexible prefrontal fNIRS headband featuring long–short channel multiplexing and dual short-channel measurement [31]. EEG signals were sampled at 250 Hz, whereas fNIRS signals were sampled at 12.5 Hz.

The fNIRS subsystem employed four 735/805/850 nm LED light sources and four silicon photomultipliers (SiPMs), forming six long channels with a source–detector distance of 30 mm and four short channels with a source–detector distance of 8.4 mm. Each long channel could be denoised using two neighboring short channels, achieving a long-short-channel optode proportion of 100% and improving the removal of superficial physiological noise. In our previous study, this fNIRS headband was experimentally validated in terms of both its dual short-channel denoising performance and its ability to distinguish mental fatigue states in the simulated driving task. The average signal-to-noise ratio of long channels was 64 dB, and the minimum dynamic optical range of short channels was 122 dB.

On the basis of this fNIRS configuration, three Ag/AgCl active EEG electrodes were added at Fp1, Fpz, and Fp2 according to the international 10–20 system. An ear-clip electrode placed at the left earlobe (A1) served as the reference electrode for the three prefrontal recording electrodes. To improve conductivity, all EEG electrodes were applied with a medical conductive paste (GT5, GREENTEK, Wuhan, China). According to the manufacturer, the paste has a conductivity of 10.0–85.0 mS/cm and an impedance of approximately 5 kΩ, allowing stable use for 4–6 h. The capability of the headband to measure spontaneous EEG rhythms was verified in an eyes-open/eyes-closed experiment. Further validation details of the headband are provided in Appendix A.

#### 2.4.2. EEG Signal Processing and Feature Extraction

The recorded EEG signals were first preprocessed to improve signal quality before feature extraction. To remove switching crosstalk, notch filtering was applied at 12.5 Hz, 25 Hz, 37.5 Hz, and 50 Hz. The signals were then band-pass filtered between 0.5 Hz and 30 Hz to retain the spontaneous EEG rhythm components relevant to fatigue analysis. After filtering, bad channels were manually inspected. Ocular artifacts in the three prefrontal channels (Fp1, Fpz, and Fp2) were further removed using a wavelet-assisted adaptive filter method [32].

The preprocessed EEG signals were segmented into non-overlapping 30 s windows. Segments shorter than 30 s were discarded. For each segment, the power spectral density and the spectral power within each frequency band were calculated. To characterize fatigue-related changes in prefrontal cortical activity, the relative power level (RPL) of four conventional EEG frequency bands was extracted, namely δ (0.5–4 Hz), θ (4–8 Hz), α (8–13 Hz), and β (14–30 Hz). For each band, the RPL was defined as the ratio of the band power to the sum of the powers of the four bands:

(1) $$\mathrm{RP}L_{m}=\frac{P_{m}}{P_{\delta }+P_{\theta }+P_{\alpha }+P_{\beta }},\;\;\;\;\;m\in \left\{\delta ,\;\theta ,\;\alpha ,\;\beta \right\},$$

where $P_{\delta }$, $P_{\theta }$, $P_{\alpha }$, and $P_{\beta }$ denote the spectral powers of the δ, θ, α, and β bands, respectively.

In addition, the ratio of β power to the sum of θ and α powers was calculated as:

(2) $$\eta_{\beta /\left(\theta +\alpha \right)}=\frac{P_{\beta }}{P_{\theta }+P_{\alpha }}.$$

$\eta_{\beta /\left(\theta +\alpha \right)}$ was used to characterize the relative dominance of task-engagement-related higher-frequency activity over fatigue-related lower-frequency components. For subsequent analysis, EEG features were obtained at both the channel-average level and the channel-specific level. Channel-average features were calculated by averaging the values of Fp1, Fpz, and Fp2, whereas channel-specific features were retained to examine spatial differences across the three prefrontal locations.

#### 2.4.3. fNIRS Preprocessing and Hemodynamic Feature Extraction

For the fNIRS signals, the raw voltages of both long and short channels were first corrected by subtracting the dark voltage measured under background-light conditions. The mean voltage during the first 2 s of the experiment was then used as the baseline. Assuming that the recorded voltage was proportional to light intensity, the change in optical density (Δ**OD**) was calculated as:

(3) $$∆OD=-\mathrm{lg}\left[\frac{I}{I_{baseline}}\right]=-\mathrm{lg}\left[\frac{V}{V_{baseline}}\right],$$

where I denotes the intensity of the emitted near-infrared light, and V denotes the voltage output by the detector.

After optical density conversion, the signal quality of each channel was visually inspected, and time periods with inadequate signal quality were excluded from further analysis. Motion artifacts in the Δ**OD** signals were then corrected using a hybrid motion-correction method based on spline interpolation and wavelet decomposition [33,34]. After motion correction, signal drift was modeled by third-order polynomial fitting and was subsequently removed from the original signal.

To remove superficial interference in the long-separation channels, dual short-channel denoising was applied to the optical-density signals of each long channel [31]. Specifically, the optical density change in each long channel (Δ**OD**_L_) was corrected using the corresponding neighboring short-channel measurements as follows:

(4) $$∆OD_{\mathrm{brain}}=∆OD_{L}-∆OD_{S}{\left(∆OD_{S}^{T}∆OD_{S}\right)}^{-1}∆OD_{S}^{T}∆OD_{L},$$

(5) $$∆OD_{S}=\left[\begin{array}{cc} ∆OD_{S-src} & ∆OD_{S-det} \end{array}\right],$$

where Δ**OD**_brain_ denotes the optical density change caused by brain activities, Δ**OD**_s-src_ and Δ**OD**_s-det_ denote the optical density change in short channels near the light source and detector in the long channel, respectively.

After denoising, concentration changes in oxyhemoglobin (Δ**HbO**) and deoxyhemoglobin (Δ**HbR**) were calculated using the modified Beer–Lambert law (MBLL) [31] and then low-pass filtered at 0.1 Hz.

(6) $$\left[\begin{array}{cc} ∆HbO & ∆HbR \end{array}\right]=\left[\frac{∆OD_{brain}\left(\lambda_{1}\right)}{L\left(\lambda_{1}\right)}\frac{∆OD_{brain}\left(\lambda_{2}\right)}{L\left(\lambda_{2}\right)}\frac{∆OD_{brain}\left(\lambda_{3}\right)}{L\left(\lambda_{3}\right)}\right]E^{+},$$

(7) $$E=\left[\begin{array}{ccc} \varepsilon_{HbO}\left(\lambda_{1}\right) & \varepsilon_{HbO}\left(\lambda_{2}\right) & \varepsilon_{HbO}\left(\lambda_{3}\right)\\ \varepsilon_{HbR}\left(\lambda_{1}\right) & \varepsilon_{HbR}\left(\lambda_{2}\right) & \varepsilon_{HbR}\left(\lambda_{3}\right) \end{array}\right],$$

where *λ*_1_–*λ*_3_ denote three near-infrared wavelengths of each LED, *L* denotes the mean photon pathlength calculated by multiplying the differential path factor (DPF) by the source–detector distance (*r*), *ε* denotes the molar extinction coefficient of the chromophore, **E**^+^ denotes the Moore–Penrose pseudoinverse of **E**.

The fNIRS subsystem provided six long channels, organized into three prefrontal channel pairs: S1D2 and S2D1, S2D3 and S3D2, and S3D4 and S4D3. The mean values of the two channels within each pair were used to represent the corresponding prefrontal locations Fp1, Fpz, and Fp2, respectively. Based on Δ**HbO** and Δ**HbR**, concentration changes in total hemoglobin (Δ**HbT**) and cerebral oxygen exchange (Δ**COE**) [35] were further calculated as:

(8) ∆HbT=∆HbO+∆HbR,

(9) ∆COE=∆HbR−∆HbO.

The preprocessed fNIRS signals were segmented into non-overlapping 30 s windows, and segments shorter than 30 s were discarded. For each window, the mean (*M*) of Δ**HbO**, Δ**HbR**, Δ**HbT**, and Δ**COE** were extracted as fNIRS features for subsequent analysis.

#### 2.4.4. Pulse Signal Extraction and Pulse Rate Variability Feature Calculation

Pulse-related signals were derived from the preprocessed short-channel fNIRS measurements. Using the MBLL, concentration changes in oxyhemoglobin and deoxyhemoglobin were calculated from the preprocessed short-channel optical density signals. The resulting pulse-related hemodynamic signals were then band-pass filtered between 0.5 Hz and 2.5 Hz to isolate the pulsatile component, yielding Δ**HbO**_pulse_ and Δ**HbR**_pulse_ for the four short channels, namely S1D1, S2D2, S3D3, and S4D4.

For each non-overlapping 30 s window, pulse peaks were detected from Δ**HbO**_pulse_ using the automatic multiscale-based peak detection algorithm [36] because its pulsatile waveform generally exhibited clearer peak morphology than that of Δ**HbR**_pulse_ [37]. Based on the detected pulse peaks, the inter-beat interval (IBI) time series, denoted as Δ*t*_IBI_, was obtained. Outlier values in the IBI series were removed before PRV analysis.

Two PRV features were then calculated from the cleaned IBI sequence: the standard deviation of normal-to-normal intervals (SDNN) and the root mean square of successive differences (RMSSD). SDNN and RMSSD were computed as

(10) $$SDNN=\sqrt{\frac{1}{N-1}\sum_{i=1}^{N}(∆t_{IBI}^{i}-\bar{∆t_{IBI}}{)}^{2}},$$

(11) $$RMSSD=\sqrt{\frac{1}{N-1}\sum_{i=1}^{N-1}(∆t_{IBI}^{i+1}-∆t_{IBI}^{i}{)}^{2}},$$

where *N* denotes the number of valid IBIs within the analysis window, $∆t_{IBI}^{i}$ denotes the i-th IBI, and $\bar{∆t_{IBI}}$ denotes the mean IBI within the window.

Finally, PRV features were averaged across the four short channels to obtain channel-averaged SDNN and RMSSD values for subsequent analysis.

### 2.5. Statistical Analysis

To reduce the influence of inter-subject variability on feature analysis, the hemodynamic and pulse-related features with physical units were normalized within each experiment using a 5–95% percentile-based min–max normalization method. Mean hemodynamic features were normalized to [−1, 1], whereas the pulse variability features were normalized to [0, 1]. Because this normalization was performed separately within each experiment, the normalized NonF values were used as within-paradigm reference levels and should not be interpreted as directly comparable absolute baselines between the active- and passive-fatigue paradigms.

Each feature sample was assigned a timestamp corresponding to the midpoint of its associated time window. According to the previously defined fatigue-label periods, samples were then assigned to NonF, ModF, or SevF. This resulted in a prefrontal multimodal physiological feature set with a repeated-measures structure, in which time-window-level samples were nested within participants.

To account for this repeated-measures structure and to accommodate unequal numbers of samples across fatigue levels, linear mixed-effects models (LMMs) were fitted separately for the active-fatigue and passive-fatigue tasks for each physiological feature [38,39]. In each model, fatigue level (NonF, ModF, and SevF) was treated as a categorical fixed effect, and participant was included as a random intercept. This window length was selected to reduce temporal dependence among adjacent samples while preserving sufficient coverage of short fatigue stages; in the present dataset, the shortest fatigue stage lasted 112 s, allowing multiple observations to be retained even in the shortest stage. Estimated marginal means and their 95% confidence intervals (CIs) were obtained from each model. Pairwise comparisons among fatigue levels were performed based on the model estimates, and *p* values were adjusted using the Holm method within each feature-specific analysis. A significance level of *p* < 0.05 was adopted for all statistical tests.

## 3. Results

### 3.1. Subjective and Behavioral Evidence for Fatigue Progression and Three-Level Labeling

#### 3.1.1. Subjective Fatigue Progression Assessed by F-ISA

**Figure 4:**
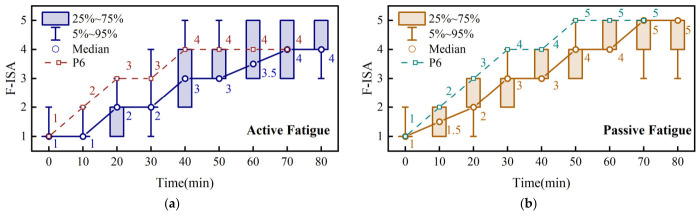
Temporal progression of subjective fatigue assessed by fatigue instantaneous self-assessment (F-ISA) during the active and passive paradigms. (**a**) Active paradigm. (**b**) Passive paradigm.

The temporal progression of subjective fatigue was evaluated using the F-ISA scores collected every 10 min during the active- and passive-fatigue tasks (Figure 4). Because the final part of P6’s physiological recordings was excluded due to poor signal quality, the corresponding late-stage subjective and behavioral data were not included in the group-level summaries, in order to maintain consistency with the subsequent physiological analyses. Thus, the first 8 F-ISA values of P6 are shown separately, whereas the scores of the other ten participants are summarized by box plots.

In both task conditions, F-ISA scores increased progressively over time, indicating that both paradigms successfully induced mental fatigue. In the active paradigm, the median F-ISA score increased from 1 at the beginning to 2 at 20–30 min, 3 at 40–50 min, and 3.5–4 during the last 20 min. In the passive paradigm, the median F-ISA score increased from 1 initially to 1.5 at 10 min, 2 at 20 min, 3 at 30–40 min, 4 at 50–60 min, and 5 at 70–80 min. The F-ISA trajectory of P6 showed a similar increasing trend in both tasks.

The Friedman test further confirmed significant stage effects in both task conditions. Specifically, the F-ISA scores varied significantly across stages in the active-fatigue task ($\chi^{2}=64.91333$, p<0.0001) and in the passive-fatigue task ($\chi^{2}=63.26667$, p<0.0001). Compared with the active-fatigue task, the passive-fatigue task showed a more gradual increase in the early stage but reached a higher subjective fatigue level in the later stage.

Overall, the F-ISA results demonstrate that subjective fatigue increased significantly and progressively in both task conditions. These results provide direct subjective evidence for the effectiveness of the two fatigue-induction paradigms and support the subsequent three-level fatigue labeling strategy.

#### 3.1.2. Facial Behavioral Changes Reflected by PERCLOS and Yawning

**Figure 5:**
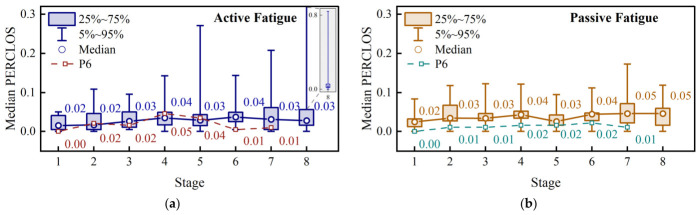

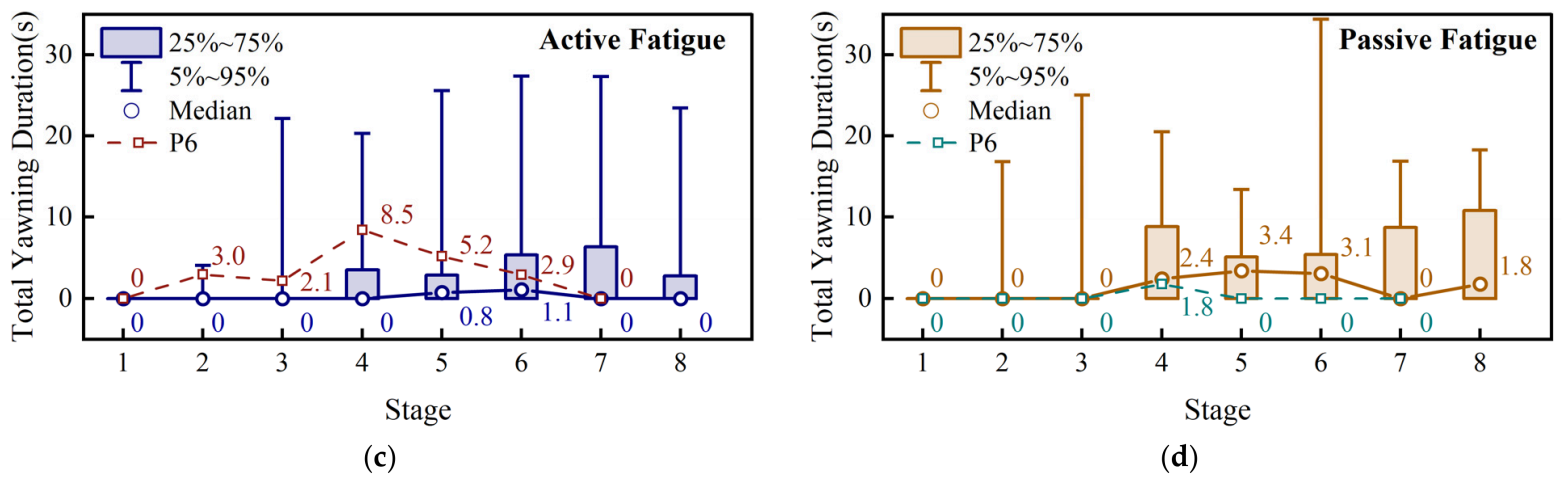
Stage-wise facial behavioral changes during the active and passive paradigms. (**a**) Median percentage of eyelid closure (PERCLOS) in the active paradigm. (**b**) Median PERCLOS in the passive paradigm. (**c**) Total yawning duration in the active paradigm. (**d**) Total yawning duration in the passive paradigm.

Fatigue-related facial behavioral changes were further examined using median PERCLOS and total yawning duration within each 10 min stage (Figure 5).

For PERCLOS, different temporal patterns were observed between the two task conditions. In the active-fatigue task, the group median of the participant-wise stage median PERCLOS values remained within a relatively low range of approximately 0.02–0.04, but the overall stage effect was significant (Friedman test, $\chi^{2}=14.53$, p=0.0426). In the passive-fatigue task, the corresponding group median showed a mild increasing trend from approximately 0.02 in the early stages to 0.05 in the later stages; however, the overall stage effect did not reach significance ($\chi^{2}=10.65$, p=0.1546). The P6 trajectory remained lower than the group-level pattern in both tasks and showed only limited fluctuations across stages.

The total yawning duration showed a sparse but fatigue-related pattern. In the active-fatigue task, the group median yawning duration remained 0 s in most stages, with only slight increases in Stages 5 and 6. In the passive-fatigue task, the group median remained 0 s during the first three stages and increased to 2.4–3.4 s during Stages 4–6, suggesting that yawning became more frequent in the middle-to-late phase of fatigue development. However, the overall stage effects were not significant in either the active-fatigue task ($\chi^{2}=9.25$, p=0.2352) or the passive-fatigue task ($\chi^{2}=11.80$, p=0.1073). Compared with the group-level pattern, P6 exhibited more pronounced yawning in the active-fatigue task, reaching 8.5 s at Stage 4, whereas only a brief increase was observed in the passive-fatigue task.

Overall, the facial behavioral measures showed fatigue-related stage trends in both task conditions, while also revealing substantial inter-subject variability. Among the examined behavioral measures, only PERCLOS in the active-fatigue task showed a significant stage effect. The absence of significant group-level effects in the other behavioral measures suggests that PERCLOS and yawning are more suitable as complementary indicators for refining fatigue-level transitions than as standalone group-level markers of fatigue progression.

#### 3.1.3. Three-Level Fatigue Labeling Results

**Figure 6:**
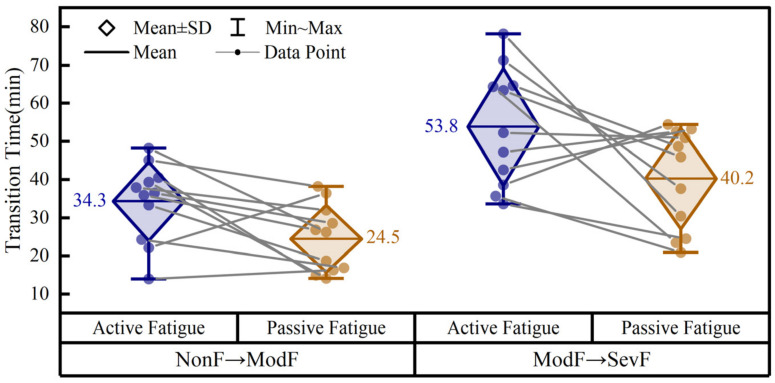
Transition times of fatigue-level progression in the active and passive paradigms. Dots represent individual participants. Gray lines connect repeated measurements from the same participant across task conditions within each transition type. The statistical effects of fatigue type and transition type were evaluated using a two-way repeated-measures ANOVA and are reported in the text.

The transition times of fatigue-level progression are summarized in Figure 6. In the active paradigm, the transition from NonF to ModF occurred at 34.3 ± 10.3 min on average, whereas the transition from ModF to SevF occurred at 53.8 ± 15.4 min. In the passive paradigm, the corresponding transition times were 24.5 ± 8.8 min and 40.2 ± 13.2 min, respectively. As shown in Figure 6, the ModF → SevF transition occurred later than the NonF → ModF transition in both task conditions, and the transition times in the passive-fatigue task were overall earlier than those in the active-fatigue task.

Before inferential analysis, the four groups of transition-time data were tested for normality using the Shapiro–Wilk test, and all groups satisfied the normality assumption (all p>0.05). A two-way repeated-measures ANOVA was then performed with fatigue type (active vs. passive) and transition type (NonF → ModF vs. ModF → SevF) as within-subject factors. The analysis revealed a significant main effect of fatigue type (F=7.82, p=0.0189). The marginal mean transition time was greater in the active-fatigue task (44.0 min, 95% CI: 36.3–51.8 min) than in the passive-fatigue task (32.3 min, 95% CI: 25.6–39.1 min), indicating that fatigue-level transitions occurred overall earlier in the passive-fatigue task. A significant main effect of transition type was also observed (F=114.34, p<0.0001). The marginal mean transition time for ModF → SevF (47.0 min, 95% CI: 40.4–53.6 min) was markedly later than that for NonF → ModF (29.4 min, 95% CI: 24.4–34.4 min). The interaction between fatigue type and transition type was not significant (F=0.41, p=0.5379), suggesting that the temporal gap between the two transition types was similar in the active- and passive-fatigue tasks.

After non-overlapping 30 s windows were generated, each window was assigned to NonF, ModF, or SevF according to the position of its midpoint relative to the fatigue-level transition times. Based on this midpoint-based labeling procedure, the numbers of labeled windows in the active-fatigue task were 755, 428, and 532 for NonF, ModF, and SevF, respectively, whereas the corresponding values in the passive-fatigue task were 539, 346, and 798.

Overall, the three-level fatigue labeling results indicate that both task conditions exhibited a progressive evolution from NonF to ModF and then to SevF. Compared with the active condition, the passive condition reached both moderate and severe fatigue earlier. These labeled windows provided the basis for the subsequent multimodal physiological feature analysis.

### 3.2. EEG Signatures Under the Active- and Passive-Fatigue-Inducing Paradigms

#### 3.2.1. Channel-Averaged EEG Features Across Fatigue Levels

**Figure 7:**
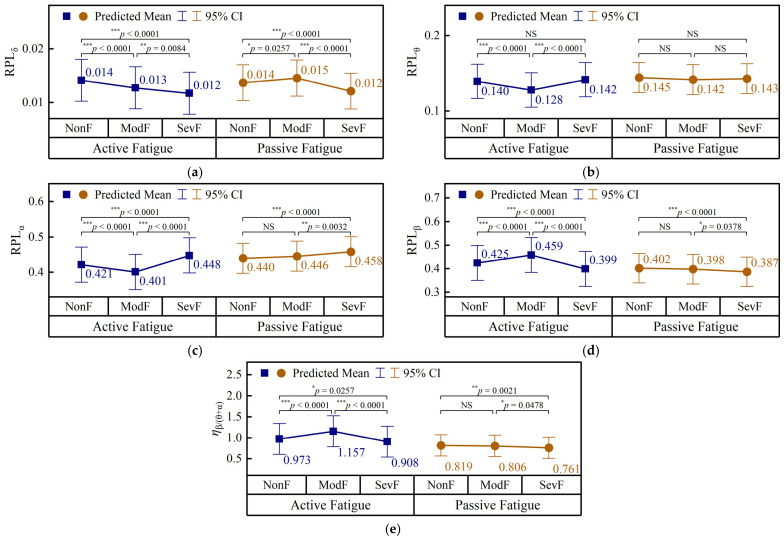
Channel-averaged EEG features across fatigue levels in the active and passive fatigue paradigms. (**a**) ${RPL}_{\delta }$; (**b**) ${RPL}_{\theta }$; (**c**) ${RPL}_{\alpha }$; (**d**) ${RPL}_{\beta }$; (**e**) $\eta_{\beta /\left(\theta +\alpha \right)}$. RPL, relative power level; $\eta_{\beta /\left(\theta +\alpha \right)}$, ratio of β power to the sum of θ and α powers; NonF, non-fatigue; ModF, moderate fatigue; SevF, severe fatigue; NS: *p *≥ 0.05; *: *p* < 0.05; **: *p* < 0.01; ***: *p* < 0.001.

The grade-related changes in channel-averaged EEG features are shown in Figure 7, where the predicted means and 95% CIs were obtained from separate LMMs for the active- and passive-fatigue tasks. Pairwise comparisons among fatigue levels were performed within each task condition, and the adjusted *p* values are indicated above the brackets.

In the active paradigm, all five EEG features showed significant fatigue-level effects. ${RPL}_{\delta }$ decreased progressively from 0.014 in NonF to 0.013 in ModF and 0.012 in SevF. By contrast, ${RPL}_{\theta }$ and ${RPL}_{\alpha }$ both decreased from NonF to ModF and then increased in SevF. ${RPL}_{\beta }$ showed the opposite pattern, increasing from 0.425 in NonF to 0.459 in ModF and then decreasing to 0.399 in SevF. The ratio $\eta_{\beta /\left(\theta +\alpha \right)}$ displayed a similar inverted-U pattern, rising from 0.973 in NonF to 1.157 in ModF and then decreasing to 0.908 in SevF.

In the passive paradigm, only ${RPL}_{\delta }$ showed a significant difference between NonF and ModF and increased from 0.014 to 0.015. When fatigue further progressed from ModF to SevF, ${RPL}_{\delta }$ decreased from 0.015 to 0.012, whereas ${RPL}_{\theta }$ remained essentially unchanged. ${RPL}_{\alpha }$ increased from 0.446 to 0.458, while ${RPL}_{\beta }$ decreased from 0.398 to 0.387. The ratio $\eta_{\beta /\left(\theta +\alpha \right)}$ also decreased markedly from 0.806 in ModF to 0.761 in SevF.

Overall, the channel-averaged EEG results indicate distinct within-task evolution patterns under the two fatigue conditions. In active fatigue, significant EEG changes were observed in both the NonF → ModF and ModF → SevF transitions, whereas in passive fatigue, the more prominent EEG alterations emerged mainly from ModF to SevF.

#### 3.2.2. Channel-Specific EEG Features Across Fatigue Levels

The channel-specific and channel-averaged changes in EEG features across fatigue levels are summarized in Table 2. Compared with the channel-averaged analysis, the channel-specific results provide additional information on the sensitivity of different prefrontal locations to early-stage fatigue changes (NonF → ModF), late-stage fatigue changes (ModF → SevF), and cumulative fatigue effects (NonF → SevF).

In the active paradigm, ${RPL}_{\alpha }$ and ${RPL}_{\beta }$ showed fully consistent patterns across all three channels. For ${RPL}_{\delta }$, only Fpz and Fp2 retained significant decreases in the ModF → SevF comparison and Fp1 did not. For ${RPL}_{\theta }$, only Fp1 showed a significant NonF → SevF increase, whereas Fpz and Fp2 did not, suggesting that the left prefrontal site were more sensitive to the cumulative fatigue effect. A similar pattern was observed for $\eta_{\beta /\left(\theta +\alpha \right)}$: only Fp1 retained a significant NonF → SevF decrease, whereas Fpz and Fp2 did not, indicating that the left prefrontal site showed relatively stronger sensitivity to cumulative changes.

In the passive paradigm, ${RPL}_{\delta }$ showed significant increases in NonF → ModF at Fp1 and Fpz, with no significant change at Fp2. By contrast, ${RPL}_{\theta }$ showed no significant changes at Fp1 or Fpz, whereas Fp2 showed significant decreases in NonF → ModF and NonF → SevF, indicating that the right prefrontal site was more sensitive to both early-stage and cumulative passive-fatigue changes. For ${RPL}_{\alpha }$, Fp1 showed an additional significant increase in NonF → ModF, whereas Fpz and Fp2 did not, suggesting that the left prefrontal site was more sensitive to early passive-fatigue changes for this feature. In the ModF → SevF comparison, Fp1 and Fpz showed significant increases, whereas Fp2 did not. For ${RPL}_{\beta }$, only Fp1 and Fpz showed significant decreases in ModF → SevF and NonF → SevF, indicating stronger sensitivity of these two sites to late-stage and cumulative changes. For $\eta_{\beta /\left(\theta +\alpha \right)}$, Fp1 showed significant decreases in all three comparisons, Fpz was sensitive mainly to late-stage and cumulative changes, whereas Fp2 showed no significant change, suggesting minimal sensitivity of the right prefrontal site for this feature under passive fatigue.

Overall, the channel-specific EEG analysis indicates that active fatigue was associated with relatively uniform spatial evolution across the three prefrontal channels, with only limited differences in cumulative sensitivity. In contrast, passive fatigue showed clearer channel dependence, with different prefrontal sites preferentially reflecting early-stage, late-stage, or cumulative fatigue-related EEG changes.

### 3.3. Hemodynamic Signatures Under the Active- and Passive-Fatigue-Inducing Paradigms

#### 3.3.1. Channel-Averaged Hemodynamic Features Across Fatigue Levels

**Figure 8:**
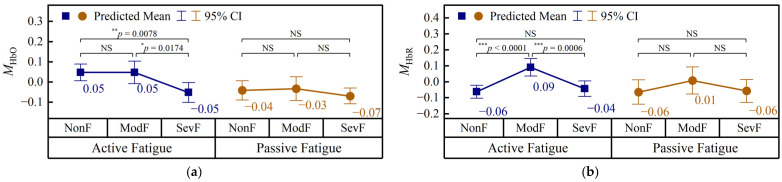

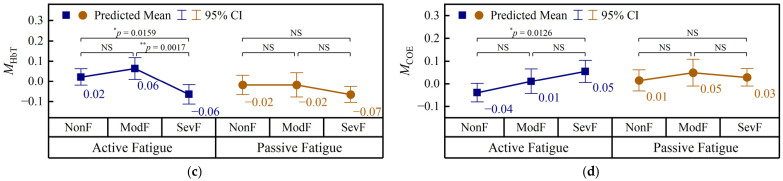
Channel-averaged hemodynamic features across fatigue levels in the active and passive paradigms. (**a**) $M_{HbO}$; (**b**) $M_{HbR}$; (**c**) $M_{HbT}$; (**d**) $M_{COE}$. M, mean feature value; HbO, oxyhemoglobin; HbR, deoxyhemoglobin; HbT, total hemoglobin; COE, cerebral oxygen exchange; NonF, non-fatigue; ModF, moderate fatigue; SevF, severe fatigue; NS: *p* ≥ 0.05; *: *p* < 0.05; **: *p* < 0.01; ***: *p* < 0.001.

The grade-related changes in the channel-averaged hemodynamic features are shown in Figure 8.

In the active paradigm, significant fatigue-level differences were observed for $M_{HbO}$, $M_{HbR}$, $M_{HbT}$, and $M_{COE}$. Among them, $M_{HbO}$ remained stable from 0.05 in NonF to 0.05 in ModF and then decreased to −0.05 in SevF, with significant differences observed for both ModF → SevF and NonF → SevF, indicating a late-stage and cumulative reduction with fatigue progression. By contrast, $M_{HbR}$ showed significant adjacent-stage changes, increasing from −0.06 in NonF to 0.09 in ModF and then decreasing to −0.04 in SevF, so that both NonF → ModF and ModF → SevF were significant whereas the NonF → SevF comparison was not. $M_{HbT}$ showed a similar non-monotonic pattern, increasing from 0.02 in NonF to 0.06 in ModF and then decreasing to −0.06 in SevF; accordingly, significant differences were observed for ModF → SevF and NonF → SevF, whereas NonF → ModF was not significant. For $M_{COE}$, the values increased from −0.04 in NonF to 0.01 in ModF and further to 0.05 in SevF, but only the NonF → SevF comparison reached significance, indicating a cumulative increase across fatigue progression.

In the passive paradigm, none of the four channel-averaged hemodynamic features showed significant fatigue-level differences. Specifically, $M_{HbO}$ changed only slightly from −0.04 in NonF to −0.03 in ModF and −0.07 in SevF, $M_{HbR}$ fluctuated from −0.06 to 0.01 and back to −0.06, $M_{HbT}$ remained nearly unchanged from −0.02 in NonF to −0.02 in ModF before decreasing slightly to −0.07 in SevF, and $M_{COE}$ changed from 0.01 to 0.05 and then to 0.03. None of these pairwise comparisons reached significance.

Overall, the channel-averaged hemodynamic results indicate that active fatigue was associated with clearer and more diverse prefrontal hemodynamic modulations than passive fatigue. In particular, active fatigue involved a late-stage and cumulative decrease in HbO, a stage-dependent fluctuation in HbR and HbT, and a cumulative increase in COE, whereas passive fatigue showed non-significant hemodynamic modulation across fatigue levels.

#### 3.3.2. Channel-Specific Hemodynamic Features Across Fatigue Levels

The channel-specific and channel-averaged hemodynamic changes are summarized in Table 3. Compared with the EEG features, the hemodynamic features showed stronger spatial heterogeneity across Fp1, Fpz, and Fp2. In addition to channel-averaged trends, the channel-specific results revealed both lateralized sensitivity and locally selective responses across channels.

In the active paradigm, some channel-averaged effects were mainly driven by one channel. For $M_{HbO}$, the channel-averaged decrease was observed in both ModF → SevF and NonF → SevF, and the channel-specific results showed that these effects were mainly reflected at Fp2, whereas Fp1 and Fpz did not show significant changes. For $M_{HbR}$, the increase from NonF → ModF and the decrease from ModF → SevF observed in the channel-averaged analysis were mainly contributed by Fp1, while Fpz and Fp2 remained non-significant. $M_{HbT}$ showed a similar pattern to $M_{HbO}$, with significant decreases in ModF → SevF and NonF → SevF at the channel-averaged level that were mainly driven by Fp2. For $M_{COE}$, the channel-averaged analysis showed a significant increase only for NonF→SevF, whereas the channel-specific results indicated a significant increase at Fp1 in NonF → ModF and at Fp2 in both ModF → SevF and NonF → SevF, suggesting different local contributions across fatigue stages.

In the passive paradigm, the channel-averaged results were entirely non-significant, but several localized effects were still observed. For $M_{HbO}$, Fp2 showed a significant decrease in NonF → ModF, whereas Fp1 and Fpz did not. For $M_{HbR}$, Fp1 showed a significant increase in NonF → ModF and a significant decrease in ModF → SevF, while Fpz and Fp2 remained non-significant throughout. $M_{HbT}$ showed a partially similar pattern to $M_{HbO}$, with Fp2 exhibiting a significant decrease in NonF → ModF, while Fp1 showed a significant increase in ModF → SevF. For $M_{COE}$, Fp2 showed significant increases only in NonF → ModF, whereas no significant changes were observed at Fp1 or Fpz.

Overall, the channel-specific hemodynamic results indicate that prefrontal oxygenation dynamics were strongly location-dependent. In active fatigue, several channel-averaged effects were mainly contributed by specific lateral sites, particularly Fp2 for $M_{HbO}$ and $M_{HbT}$, and Fp1 for $M_{HbR}$. In passive fatigue, although the channel-averaged changes were non-significant, localized channel-specific effects were still present, again mainly at Fp1 or Fp2, suggesting that passive-fatigue-related hemodynamic modulation was weaker at the global level but remained spatially selective.

### 3.4. Pulse Variability Signatures Under the Active- and Passive-Fatigue-Inducing Paradigms

**Figure 9:**
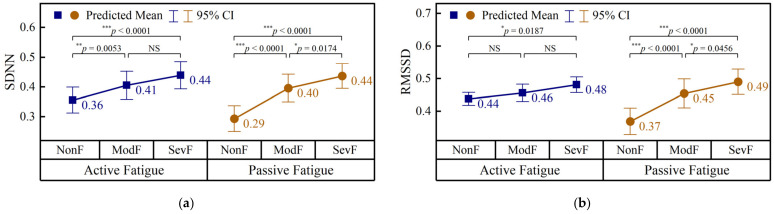
Channel-averaged pulse variability features across fatigue levels in the active and passive paradigms. (**a**) Standard deviation of normal-to-normal intervals (SDNN); (**b**) root mean square of successive differences (RMSSD). NonF, non-fatigue; ModF, moderate fatigue; SevF, severe fatigue; NS: *p* ≥ 0.05; *: *p* < 0.05; **: *p* < 0.01; ***: *p* < 0.001.

The grade-related changes in the channel-averaged pulse variability features are shown in Figure 9.

In the active paradigm, both SDNN and RMSSD showed significant fatigue-level differences. SDNN increased progressively from 0.36 in NonF to 0.41 in ModF and 0.44 in SevF, with significant differences observed only for NonF → ModF and NonF → SevF. Similarly, RMSSD increased from 0.44 in NonF to 0.46 in ModF and 0.48 in SevF. In this case, only the NonF → SevF comparison was significant, whereas the NonF → ModF and ModF → SevF comparisons were not significant.

In the passive paradigm, the two PRV features showed similar monotonic increasing trends. SDNN increased from 0.29 in NonF to 0.40 in ModF and 0.44 in SevF, and all three pairwise comparisons were significant. RMSSD also increased progressively from 0.37 in NonF to 0.45 in ModF and 0.49 in SevF, with significant differences observed for all three pairwise comparisons.

Overall, both active and passive fatigue were associated with increased pulse variability, as reflected by the significant increases in SDNN and RMSSD. The increase was more gradual in active fatigue, whereas passive fatigue showed clearer monotonic growth across fatigue levels. These findings suggest that PRV features were sensitive to fatigue progression under both task conditions and may reflect autonomic regulation changes accompanying mental fatigue.

## 4. Discussion

The present study investigated two simulated driving paradigms designed to induce active and passive fatigue. We examined whether these paradigms exhibit distinct prefrontal multimodal physiological evolution patterns when fatigue is divided into three levels—NonF, ModF, and SevF—rather than treated as a simple binary contrast. This three-level framework is an important feature of the present work, because much of the previous literature has mainly compared two states, such as well-rested vs. sleep-deprived or alert vs. fatigued, or has described fatigue development only as a continuous time-on-task effect [10,30,40]. In contrast, the present design allowed us to distinguish features sensitive mainly to the early transition (NonF → ModF), the late transition (ModF → SevF), or the cumulative contrast (NonF → SevF). Overall, the active-fatigue-inducing paradigm showed stronger and more coordinated multimodal modulation, whereas the passive-fatigue-inducing paradigm showed weaker averaged hemodynamic effects, greater spatial heterogeneity, and clearer autonomic accumulation.

### 4.1. Stage-Dependent EEG Signatures Under the Active- and Passive-Fatigue-Inducing Paradigms

EEG was the most sensitive modality for characterizing fatigue progression, especially in active fatigue. In the active paradigm, several EEG features changed significantly across both NonF → ModF and ModF → SevF, indicating continuous cortical reorganization as fatigue deepened. In contrast, in the passive paradigm, EEG changes were generally weaker in the early stage and became more evident mainly in ModF → SevF, suggesting a slower development of cortical disengagement under monotonous low-load conditions. This overall pattern is consistent with previous studies showing that EEG is highly sensitive to mental-fatigue development under time-on-task or passive-like conditions, such as the gradual increase in parietal alpha and frontal theta in a low, constant-load simulated flight task and the increase in alpha power during passive fatigue in automated monotonous driving [10,40].

The present study extends these previous mostly two-level or time-course designs by showing that EEG features do not simply differentiate “fatigued” from “non-fatigued”, but may preferentially reflect early, late, or cumulative fatigue changes. In particular, the non-monotonic trajectories observed in active fatigue suggest that the moderate-fatigue stage may reflect a compensatory-like state, in which prefrontal control may still be relatively more engaged, before shifting toward a more fatigued state. This stage-dependent view is difficult to capture in binary fatigue paradigms such as the well-rested vs. sleep-deprived design and therefore represents an important contribution of the present three-level framework [30].

### 4.2. Neurovascular Patterns and Spatial Heterogeneity of Prefrontal Hemodynamics

Compared with EEG, the hemodynamic features showed a more complex and task-dependent pattern. In the active paradigm, the late-stage and cumulative decrease in HbO, the non-monotonic fluctuation in HbR and HbT, and the cumulative increase in COE suggest a growing oxygen supply–demand imbalance in the prefrontal cortex as fatigue progressed. This differs from some previous demanding-task studies, such as Chuang et al. (2018), who reported increasing HbO during prolonged attention-demanding driving and interpreted it as a fatigue-fighting response [21]. Taken together, these findings suggest that prefrontal hemodynamic responses during fatigue do not follow a single direction, but may depend on the balance between compensatory activation, metabolic burden, and vascular adaptation. This interpretation is also compatible with the broader fNIRS literature reviewed by Yan et al. (2025), which highlighted substantial heterogeneity in fatigue-related prefrontal hemodynamic responses across paradigms [4].

A more distinctive result emerged in the passive paradigm. Although the channel-averaged hemodynamic features were non-significant, the channel-specific analysis revealed localized and channel-selective sensitivity, indicating substantial spatial heterogeneity. This suggests that the passive-fatigue-inducing paradigm was associated with localized and spatially non-uniform vascular regulation, with meaningful local changes being partly canceled after channel averaging. This observation is not emphasized in most previous studies, which have typically relied on two-level contrasts or region-averaged analyses, and therefore represents one of the novel findings of the present work.

The localized hemodynamic responses in the passive-fatigue-inducing paradigm may reflect spatially selective prefrontal regulation during sustained monotonous monitoring. Because passive driving involved lower external stimulation but prolonged vigilance demands, fatigue-related vascular changes may not appear as uniform prefrontal modulation but rather as localized channel-specific responses. The concurrent increase in pulse variability suggests that fatigue progression also involved systemic autonomic regulation. Therefore, localized hemodynamic responses and pulse variability changes may provide complementary information on fatigue development. However, the present study did not directly model neurovascular–autonomic coupling, and the limited prefrontal channel coverage does not support a definitive conclusion regarding hemispheric laterality.

### 4.3. Pulse Variability as a Stable Marker of Fatigue Accumulation

The pulse variability features showed the clearest monotonic trend across fatigue levels, particularly in passive fatigue. In both driving paradigms, SDNN and RMSSD increased with fatigue progression, with an especially clear monotonic trend in passive fatigue. This suggests that autonomic regulation was sensitive to fatigue accumulation regardless of fatigue type. This finding is partly in line with the broader mental-fatigue literature. For example, Goodman et al. (2025) reported in their meta-analysis that mental fatigue was associated with significant changes in autonomic indices, including RMSSD, although the direction of some HRV-related measures varied across studies and paradigms [2].

The present study adds that, under a three-level fatigue framework, pulse variability was able to reflect not only the final NonF → SevF contrast, but also the progressive accumulation of fatigue severity. In particular, pulse variability changed clearly in passive fatigue even when averaged hemodynamic effects were weak. This suggests that autonomic markers may provide a more robust indicator of fatigue accumulation. In contrast, EEG and hemodynamic features may be more informative about fatigue mechanism and fatigue stage. Practically, this supports the use of pulse variability as a stable component in multimodal fatigue-monitoring systems.

### 4.4. Practical Implications

Practically, these findings may inform the development of graded fatigue-monitoring systems in driving. Compared with binary fatigue detection, the three-level framework may allow earlier identification of moderate fatigue and more refined recognition of severe fatigue. EEG and hemodynamic features may provide information about task-dependent prefrontal regulation, whereas pulse variability features may serve as relatively stable indicators of fatigue accumulation. Therefore, multimodal prefrontal monitoring may support fatigue assessment in both cognitively demanding and monotonous driving scenarios and may contribute to future warning systems that adapt intervention strategies according to fatigue stage and driving condition.

### 4.5. Limitations

Several limitations should be acknowledged. First, the fatigue labels were established from subjective ratings and facial behavioral indicators, which may introduce uncertainty in the exact transition timing between fatigue levels. Second, the observed differences should be interpreted as paradigm-level differences associated with active- and passive-fatigue induction, rather than as a pure isolated effect of fatigue type alone. Third, only prefrontal signals were analyzed, so the present results do not capture possible fatigue-related changes in other cortical or systemic regions. Fourth, the participant sample was relatively homogeneous, and future validation in larger, more diverse, and mixed-sex populations is still needed. Fifth, because active- and passive-fatigue features were normalized separately, the present analyses mainly support comparisons of within-task evolution patterns rather than direct absolute contrasts between the two paradigms. In addition, the order of the two driving paradigms was not randomized; therefore, potential order effects cannot be completely excluded, although the two sessions were separated by at least 24 h. Finally, sleep duration and sleep quality before the experimental days were not quantitatively recorded. Future studies should include more systematic pre-experiment sleep assessment.

In summary, the present study shows that prefrontal multimodal physiological responses to fatigue are shaped not only by fatigue type but also by fatigue stage. Relative to the predominantly two-level literature, the present three-level design reveals that some markers mainly reflect the cumulative contrast between NonF and SevF, whereas others preferentially capture the early (NonF → ModF) or late (ModF → SevF) evolution of fatigue. Future studies should further examine whether this stage-sensitive multimodal framework can improve earlier fatigue detection and more refined fatigue-state monitoring in practical driving scenarios.

## 5. Conclusions

This study investigated the prefrontal multimodal physiological patterns under two simulated driving paradigms designed to induce active and passive fatigue by using EEG, hemodynamic, and pulse variability features acquired from a wearable prefrontal sensing system. By dividing fatigue into three levels (NonF, ModF, and SevF), the results showed that the two paradigms were associated with distinct stage-dependent evolution patterns. Specifically, the active-fatigue-inducing paradigm was associated with clearer and more continuous EEG changes across both fatigue transitions and more evident channel-averaged hemodynamic modulation, whereas the passive-fatigue-inducing paradigm showed weaker average hemodynamic effects but substantial spatial heterogeneity and clearer autonomic accumulation. In both task conditions, pulse variability features generally increased with fatigue level, indicating that they were sensitive to fatigue accumulation across both paradigms. Compared with the commonly used two-level framework in previous studies, the present three-level design further revealed that different physiological features were preferentially sensitive to the early transition (NonF → ModF), the late transition (ModF → SevF), or the cumulative contrast (NonF → SevF). These findings support the value of multimodal prefrontal monitoring for a more refined characterization of fatigue progression in simulated driving.

## Figures and Tables

**Table 1:** Comparison of the active- and passive-fatigue driving paradigms.

Category	Active-Fatigue Driving Task	Passive-Fatigue Driving Task
Vehicle	Truck tractor without trailer	Car
Route	Circular route around Corsica	Munich–Warsaw–Leipzig
Road type	Two-way, two-lane rural road	Two-way, eight-lane highway
Cruise control	Closed	Open
Cognitive workload level	High	Low
Driving time	80 min	80 min

**Table 2:** Channel-specific and channel-averaged changes in EEG features across fatigue levels under active- and passive-fatigue tasks.

Feature	Task	NonF → ModF				ModF → SevF				NonF → SevF			
		Fp1	Fpz	Fp2	ChAvg	Fp1	Fpz	Fp2	ChAvg	Fp1	Fpz	Fp2	ChAvg
${RPL}_{\delta }$	AF	↓	↓	↓	↓	NS	↓	↓	↓	↓	↓	↓	↓
	PF	↑	↑	NS	↑	↓	↓	↓	↓	↓	↓	↓	↓
${RPL}_{\theta }$	AF	↓	↓	↓	↓	↑	↑	↑	↑	↑	NS	NS	NS
	PF	NS	NS	↓	NS	NS	NS	NS	NS	NS	NS	↓	NS
${RPL}_{\alpha }$	AF	↓	↓	↓	↓	↑	↑	↑	↑	↑	↑	↑	↑
	PF	↑	NS	NS	NS	↑	↑	NS	↑	↑	↑	↑	↑
${RPL}_{\beta }$	AF	↑	↑	↑	↑	↓	↓	↓	↓	↓	↓	↓	↓
	PF	↓	NS	NS	NS	↓	↓	NS	↓	↓	↓	NS	↓
$\eta_{\beta /\left(\theta +\alpha \right)}$	AF	↑	↑	↑	↑	↓	↓	↓	↓	↓	NS	NS	↓
	PF	↓	NS	NS	NS	↓	↓	NS	↓	↓	↓	NS	↓

AF: active fatigue; PF: passive fatigue; NonF, non-fatigue; ModF, moderate fatigue; SevF, severe fatigue; ChAvg: channel-averaged; NS: not significant (*p* ≥ 0.05); ↑ indicates a significant (*p* < 0.05) increase between two fatigue levels, and ↓ indicates a significant (*p* < 0.05) decrease.

**Table 3:** Channel-specific and channel-averaged changes in hemodynamic features across fatigue levels under active- and passive-fatigue tasks.

Feature	Task	NonF → ModF				ModF → SevF				NonF → SevF			
		Fp1	Fpz	Fp2	ChAvg	Fp1	Fpz	Fp2	ChAvg	Fp1	Fpz	Fp2	ChAvg
$M_{HbO}$	AF	NS	NS	NS	NS	NS	NS	↓	↓	NS	NS	↓	↓
	PF	NS	NS	↓	NS	NS	NS	NS	NS	NS	NS	NS	NS
$M_{HbR}$	AF	↑	NS	NS	↑	↓	NS	NS	↓	NS	NS	NS	NS
	PF	↑	NS	NS	NS	↓	NS	NS	NS	NS	NS	NS	NS
$M_{HbT}$	AF	NS	NS	NS	NS	NS	NS	↓	↓	NS	NS	↓	↓
	PF	NS	NS	↓	NS	↓	NS	NS	NS	NS	NS	NS	NS
$M_{COE}$	AF	↑	NS	NS	NS	NS	NS	↑	NS	NS	NS	↑	↑
	PF	NS	NS	↑	NS	NS	NS	NS	NS	NS	NS	NS	NS

AF: active fatigue; PF: passive fatigue; ChAvg: channel-averaged; NonF, non-fatigue; ModF, moderate fatigue; SevF, severe fatigue; NS: not significant (*p* ≥ 0.05); ↑ indicates a significant (*p* < 0.05) increase between two fatigue levels, and ↓ indicates a significant (*p* < 0.05) decrease.

**Figure A1:**
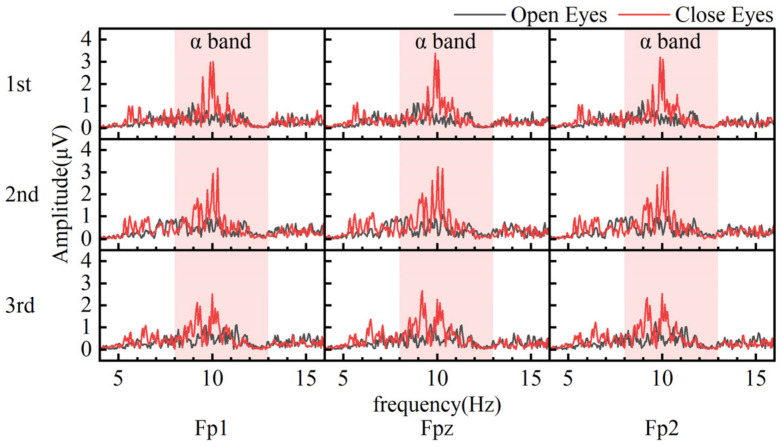
Amplitude spectra of the three prefrontal EEG channels during alternating eyes-open and eyes-closed conditions for participant 1.

**Figure A2:**
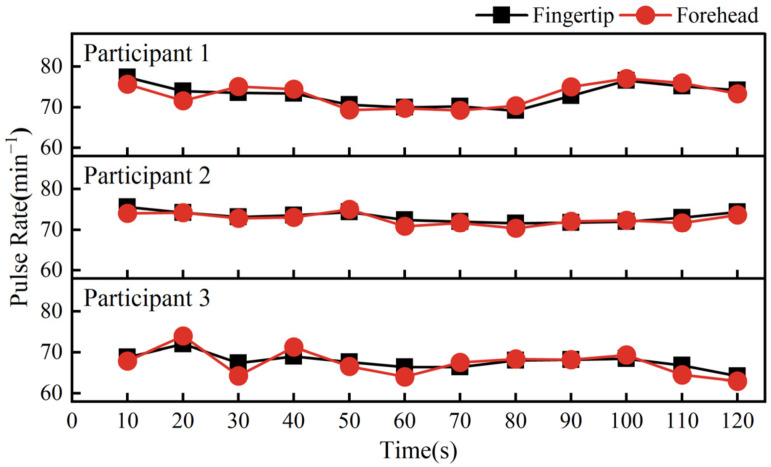
Comparison of pulse-rate traces measured at the forehead and left index finger during a 2 min seated resting condition.

## Data Availability

The data presented in this study are available on request from the corresponding authors. The data are not publicly available due to privacy restrictions.

## Abbreviations

The following abbreviations are used in this manuscript:

EEG	Electroencephalography
fNIRS	Functional near-infrared spectroscopy
PRV	Pulse rate variability
NonF	Non-fatigue
ModF	Moderate fatigue
SevF	Severe fatigue
F-ISA	Fatigue instantaneous self-assessment
PERCLOS	Percentage of eyelid closure
RPL	Relative power level
HbO	Oxyhemoglobin
HbR	Deoxyhemoglobin
HbT	Total hemoglobin
COE	Cerebral oxygen exchange
IBI	Inter-beat interval
SDNN	Standard deviation of normal-to-normal intervals
RMSSD	Root mean square of successive differences
LMM	Linear mixed-effects model
CI	Confidence interval
AF	Active fatigue
PF	Passive fatigue
ChAvg	Channel-averaged
NS	Not significant

## Appendix A. Validation of EEG and Pulse Detection Performance

### Appendix A.1. EEG Detection Validation

To further verify the EEG acquisition capability of the self-developed prefrontal EEG-fNIRS headband based on our previous study [31], additional bench and human-subject tests were conducted.

First, the input-referred noise of the EEG acquisition channel was evaluated by shorting the recording electrode and the reference electrode and recording the output voltage for 1 min. The root mean square value $V_{rms}$ and peak-to-peak value $V_{pp}$ of the output voltage were calculated to characterize the baseline noise level. In addition, the dynamic range (DR) of the EEG acquisition channel was assessed. The obtained EEG performance metrics are summarized in Table A1. The measured values were close to the reference specifications provided in the datasheet of the ADS1299 analog front end used in the acquisition board, indicating that the developed system achieved EEG acquisition performance comparable to the expected hardware level.

**Table A1:** EEG detection performance of the self-developed prefrontal EEG-fNIRS headband.

Performance Metrics	Fp1	Fpz	Fp2	ADS1299
$V_{rms}$/μV	0.49	0.44	0.42	0.32
$V_{pp}$/μV	5.32	4.25	3.62	2.26
DR/dB	115	116	116	118

Second, an eyes-open/eyes-closed experiment was performed in three healthy participants to verify whether the system could capture spontaneous EEG rhythm changes. Each participant alternated between 20 s eyes-open and 20 s eyes-closed states for three cycles while prefrontal EEG signals were recorded from Fp1, Fpz, and Fp2. The amplitude spectra of the three EEG channels for participant 1 are shown in Figure A1. Compared with the eyes-open condition, the eyes-closed condition exhibited a clearer enhancement in the α-band activity, demonstrating that the developed headband was able to detect the expected EEG rhythmic modulation associated with eye closure.

To further quantify this difference, the maximum alpha-band amplitude was identified for each trial, and the mean of the three maximum alpha amplitudes was calculated for each participant and each channel under both conditions. The results are summarized in Table A2. All three participants showed consistently higher alpha-band peak amplitudes during eye closure than during eye opening across the three prefrontal channels, although the magnitude of enhancement varied across individuals.

**Table A2:** Mean of the three α-band peak amplitudes (μV) for each participant and channel under the eye-open and eye-closed conditions.

Participant	Channel	Mean of the Three α-Band Peak Amplitudes (μV)	
		Open Eye	Close Eye
1	Fp1	1.08	2.89
	Fpz	1.13	3.09
	Fp2	1.16	2.98
	Mean ± SD	1.12 ± 0.04	2.99 ± 0.10
2	Fp1	1.00	2.49
	Fpz	1.25	2.58
	Fp2	1.03	2.60
	Mean ± SD	1.09 ± 0.14	2.56 ± 0.06
3	Fp1	1.11	1.33
	Fpz	1.22	1.79
	Fp2	1.07	1.47
	Mean ± SD	1.13 ± 0.08	1.53 ± 0.24

These results support the feasibility of the system for prefrontal EEG monitoring in the subsequent simulated driving experiments.

### Appendix A.2. Pulse Detection Validation

To validate the pulse detection capability of the developed system, comparative measurements were performed in three healthy participants during a 2 min seated resting condition.

For each participant, pulse signals were simultaneously obtained from the forehead and the left index finger. The forehead pulse signal was derived from the short-channel fNIRS measurements of the developed prefrontal headband, whereas the fingertip pulse rate (PR) was measured using a commercial finger-clip pulse oximeter (H100N, Edan, Shenzhen, China) and served as the reference. The pulse-rate traces obtained from the forehead and fingertip measurements for the three participants are shown in Figure A2.

To quantitatively evaluate the agreement between the two measurement methods, the root mean square error (RMSE) and Pearson correlation coefficient (*ρ*) were calculated between the forehead pulse rate and the fingertip pulse rate for each participant. The results are listed in Table A3. Overall, the forehead-derived pulse rate showed good consistency with the reference fingertip measurements, indicating that the developed system can reliably extract pulse-related information from prefrontal optical signals.

**Table A3:** Agreement metrics between forehead-derived pulse rate and fingertip reference pulse rate.

Performance Metrics	Position	Participant		
		1	2	3
PR/min^−1^	fingertip	73.10 ± 2.65	73.18 ± 1.27	67.77 ± 1.88
	forehead	73.09 ± 2.86	72.67 ± 1.40	67.41 ± 3.21
RMSE/min^−1^	forehead	1.37	0.88	1.72
*ρ*	forehead	0.87	0.85	0.89

These validation results support the use of the developed headband not only for prefrontal EEG and hemodynamic monitoring, but also for pulse-related physiological analysis in the main fatigue experiment.
